# Supplementary material for: Effect of a Total Extract and Saponins from Astragalus glycyphyllos L. on Human Coronavirus Replication In Vitro
Source: Int J Mol Sci. 2023 Nov 20;24(22):16525. doi: 10.3390/ijms242216525 (PMC10671514; doi:10.3390/ijms242216525)
Supplement: Supplementary file 1 [file ijms-24-16525-s001.zip › ijms-2705456-supplementary.pdf]

Supplementary

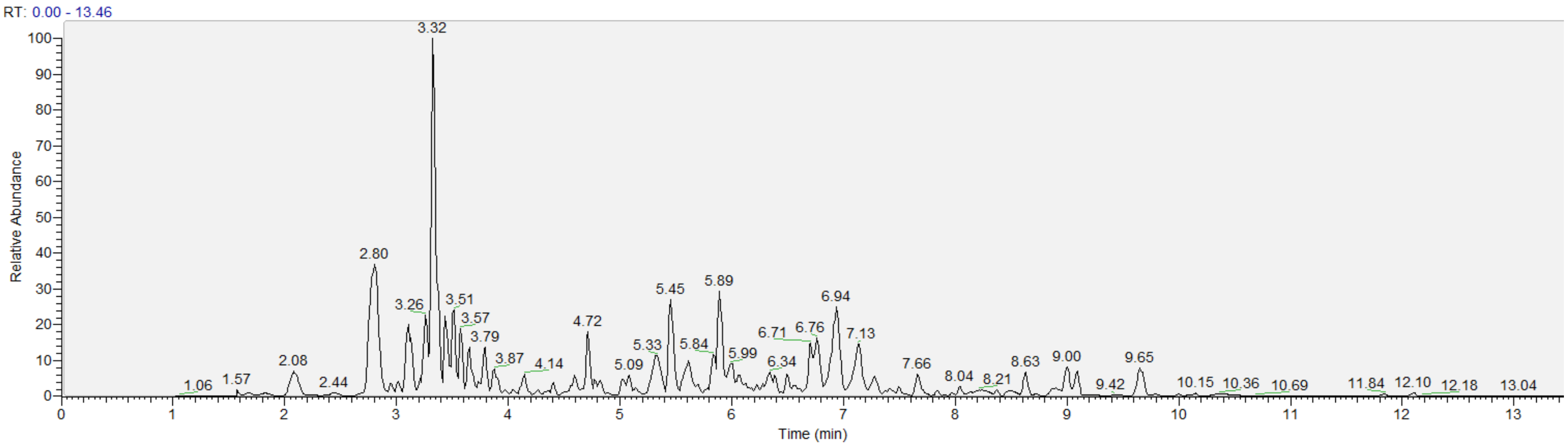

Figure S1. UHPLC-HRESIMS chromatogram (FS) of DEAG in the negative mode.

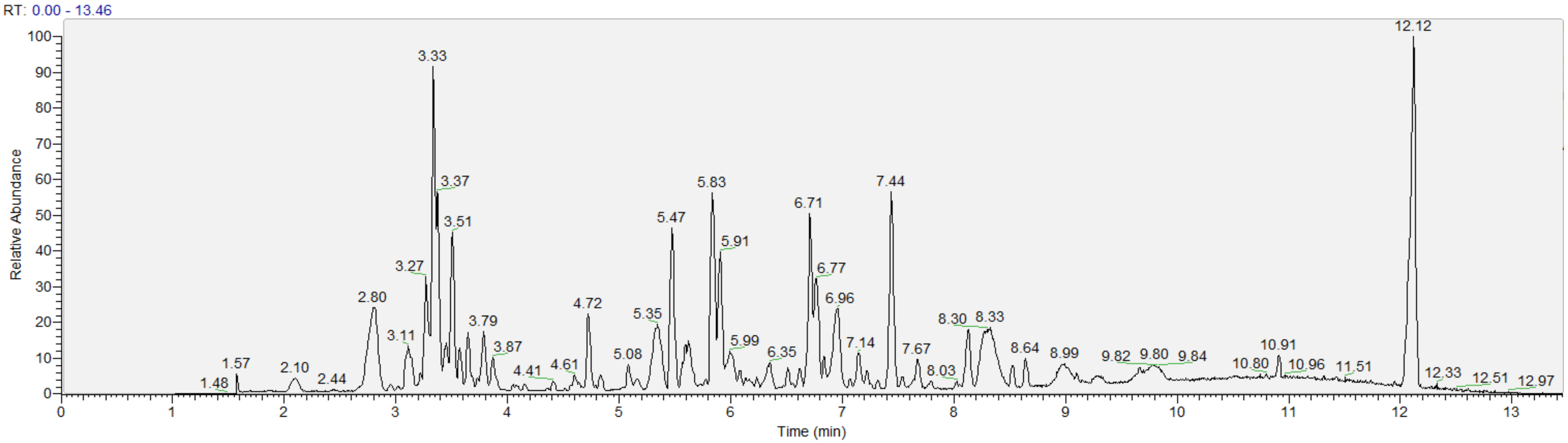

Figure S2. UHPLC-HRESIMS chromatogram (FS) of DEAG in the positive mode.

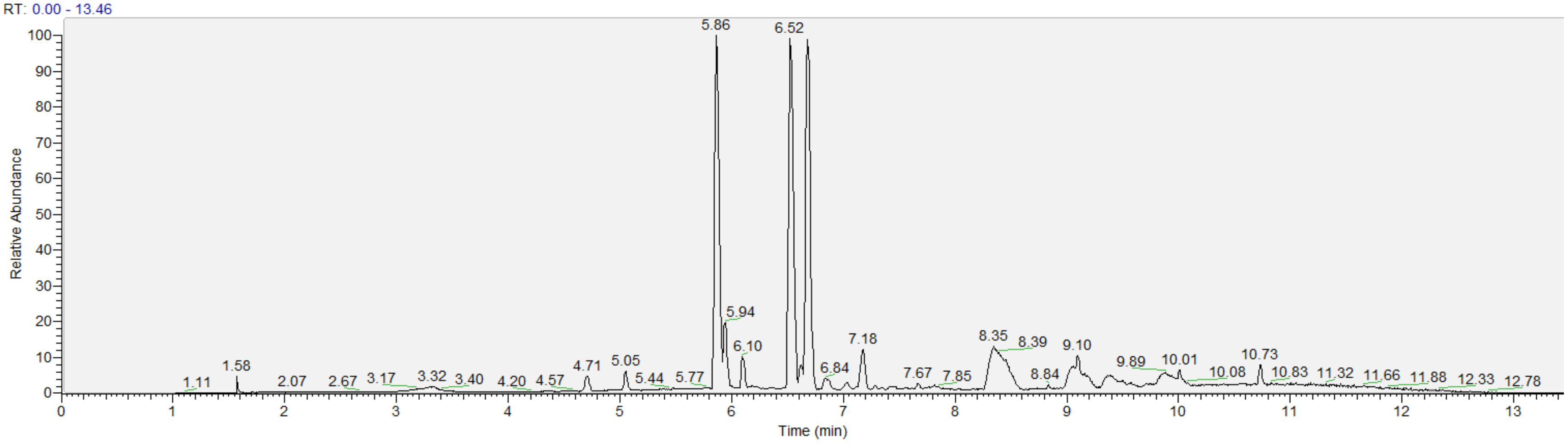

Figure S3. UHPLC-HRESIMS chromatogram (FS) of PSM in the negative mode.

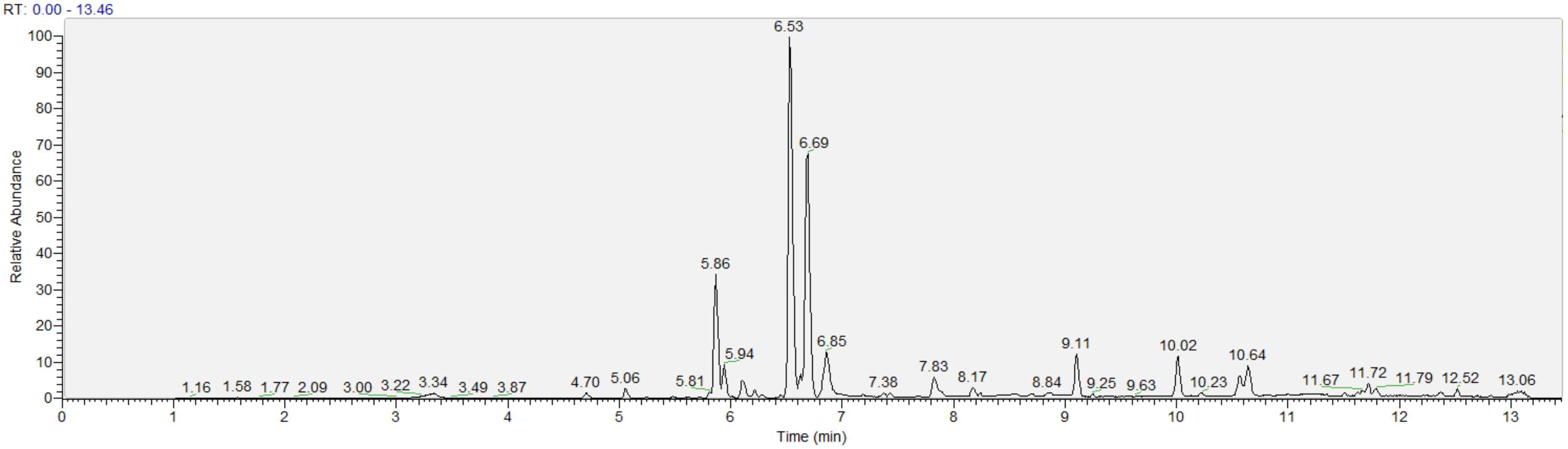

Figure S4. UHPLC-HRESIMS chromatogram (FS) of PSM in the positive mode.

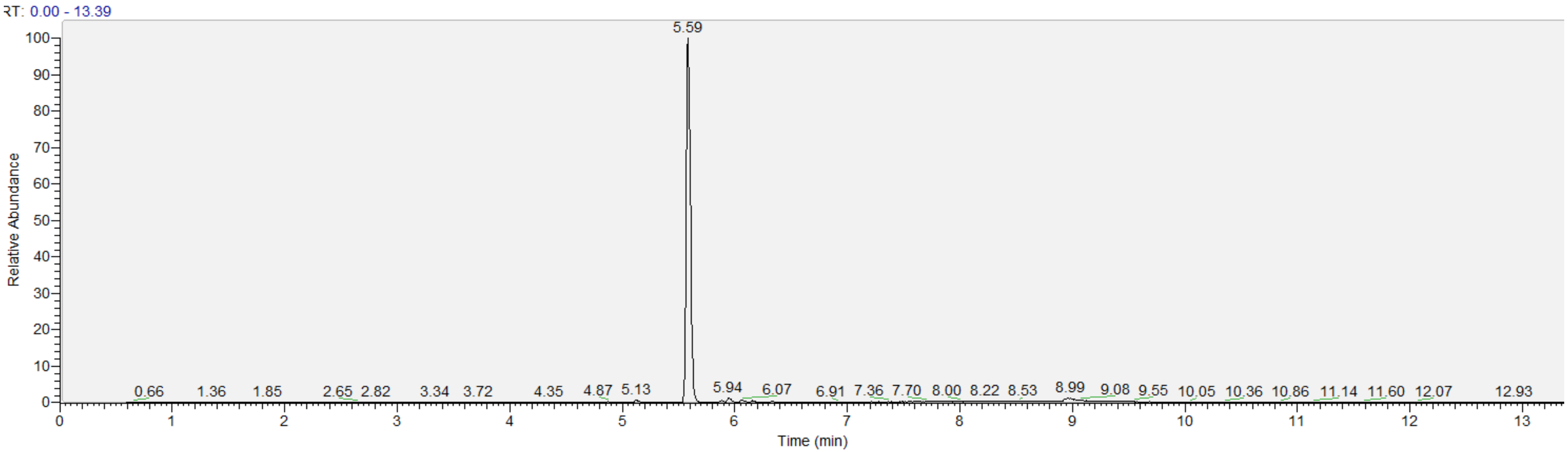

Figure S5. UHPLC-HRESIMS chromatogram (*m/z* 577.34, $C_{32}H_{49}O_9$) of S1 in the negative mode.

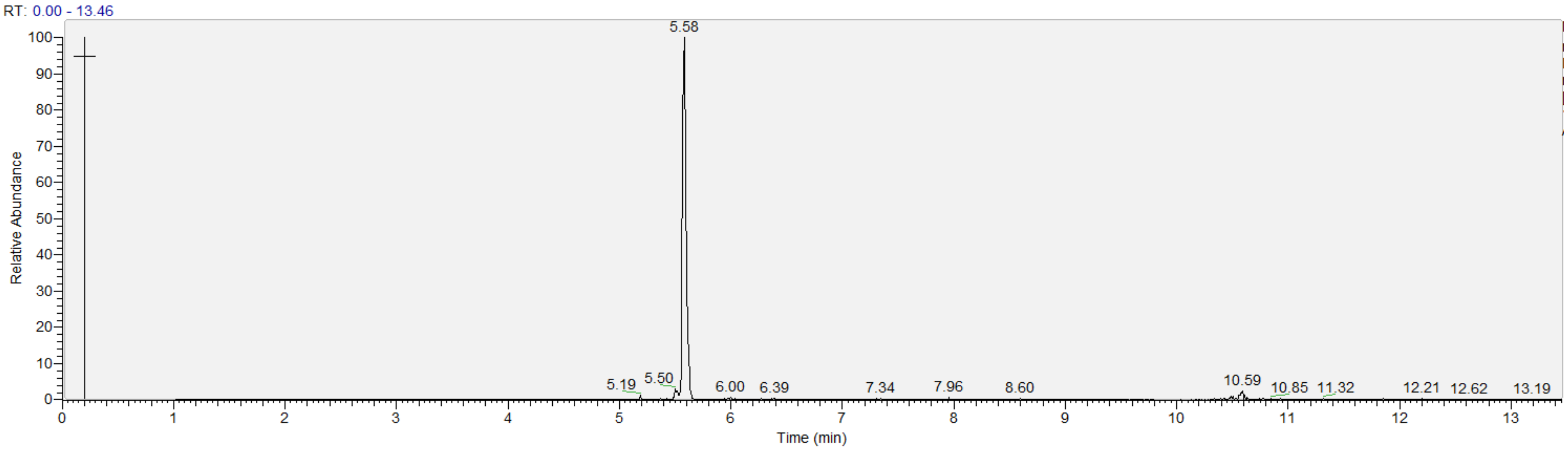

Figure S6. UHPLC-HRESIMS chromatogram (*m/z* 579.35, $C_{32}H_{51}O_9$) of S1 in the positive mode.

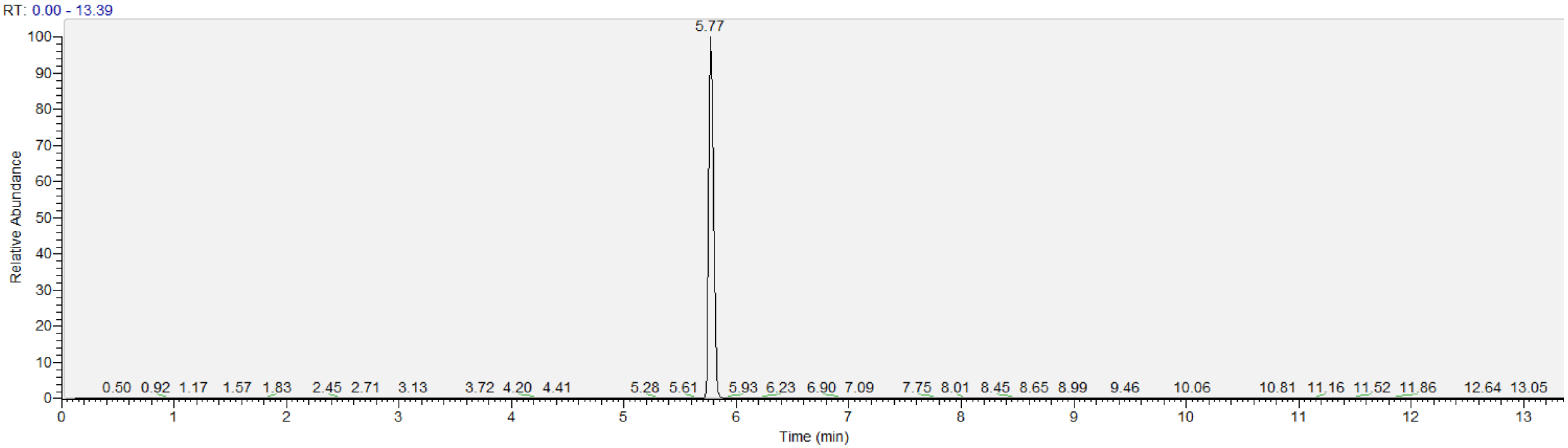

Figure S7. UHPLC-HRESIMS chromatogram (*m/z* 901.52, $C_{46}H_{77}O_{17}$) of S3 in the negative mode.

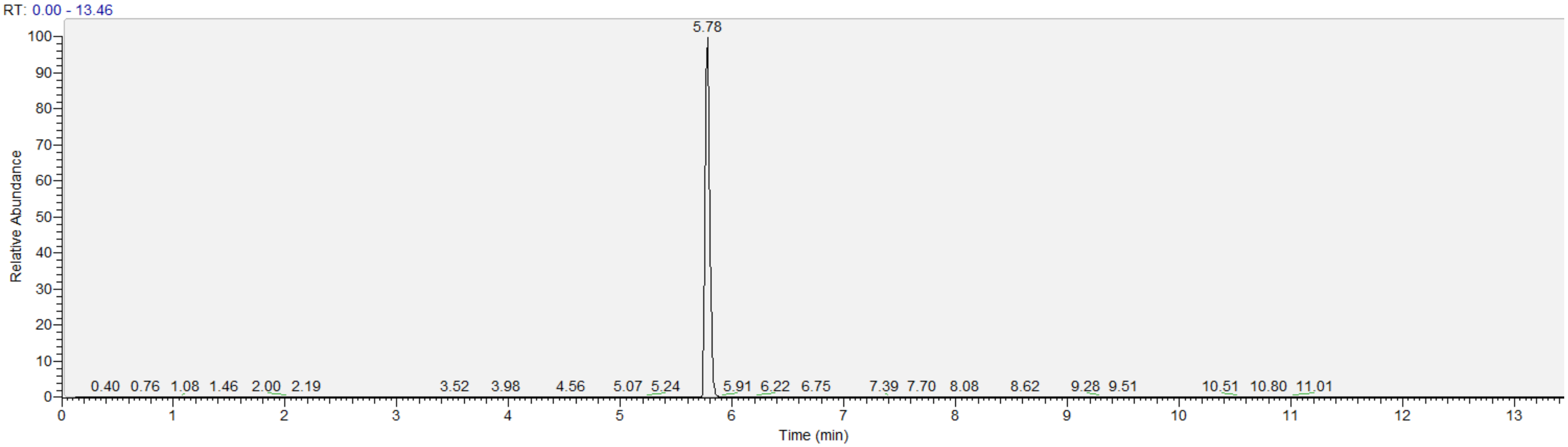

Figure S8. UHPLC-HRESIMS chromatogram (*m/z* 903.53, $C_{46}H_{79}O_{17}$) of S3 in the positive mode.

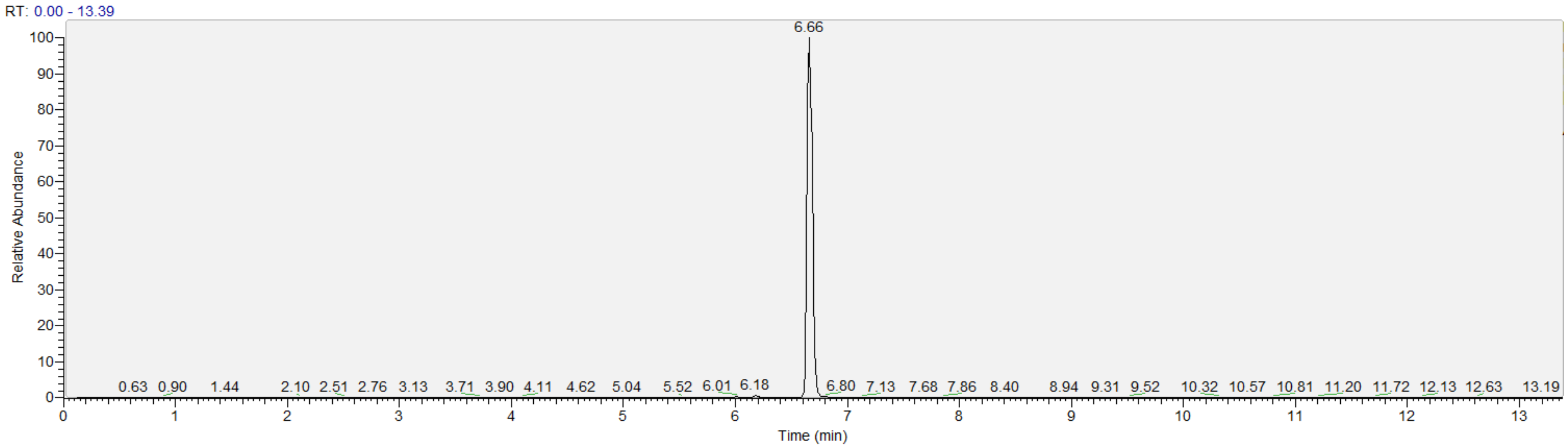

Figure S9. UHPLC-HRESIMS chromatogram (*m/z* 767.45, $C_{41}H_{67}O_{13}$) of S9 in the negative mode.

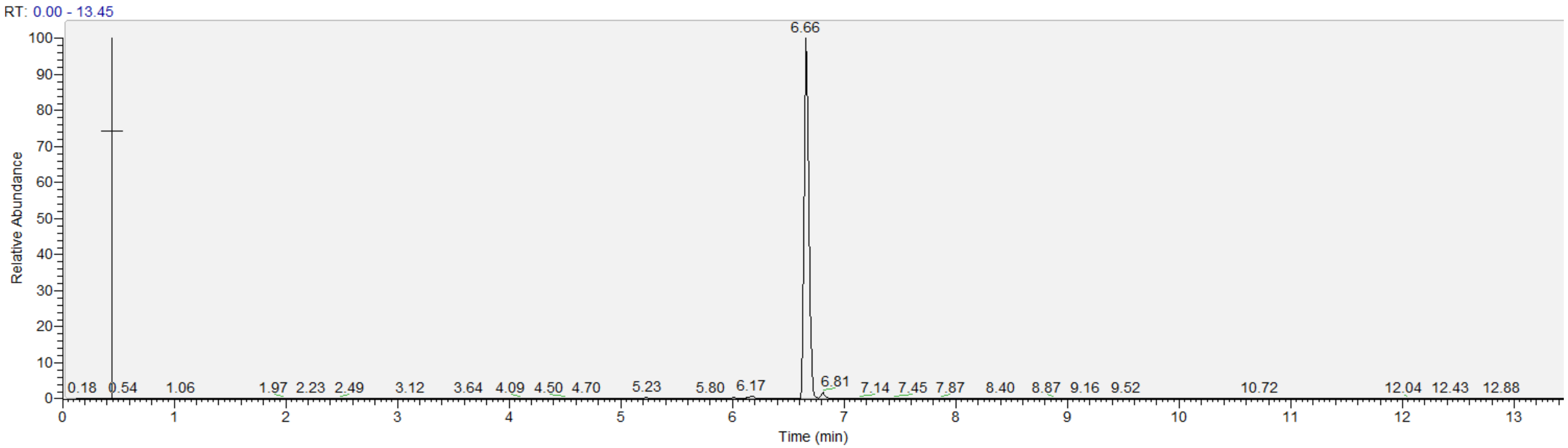

Figure S10. UHPLC-HRESIMS chromatogram (*m/z* 769.47, $C_{41}H_{69}O_{13}$) of S9 in the positive mode.
